# Effect of Indentation Load on Nanomechanical Properties Measured in a Multiphase Boride Layer

**DOI:** 10.3390/ma14216727

**Published:** 2021-11-08

**Authors:** Piotr Dziarski, Natalia Makuch

**Affiliations:** Institute of Materials Science and Engineering, Poznan University of Technology, 60-965 Poznan, Poland; piotr.dziarski@put.poznan.pl

**Keywords:** gas boriding, nanoindentation, indentation size effect, nickel borides, chromium borides

## Abstract

The study investigated the dependence of the indentation load on nanomechanical properties for a gas-borided layer produced on Inconel 600-alloy. During the measurements, the indentation load range from 10 mN to 500 mN was used. Three types of tested areas, differing in the concentration of chromium, were examined. The increase in chromium concentration was accompanied by an increase in indentation hardness and Young’s modulus. Simultaneously, the increase in the indentation load resulted in a decrease in the indentation hardness and Young’s modulus, for each type of the tested area. The presence of the indentation size effect was analyzed using four models: Meyer’s law, Hays and Kendall model, Li and Bradt model, Nix and Gao model. For all tested areas, good agreement with the Meyer’s law was obtained. However, areas with a higher chromium concentration were more susceptible to indentation size effect (ISE). The proportional specimen resistance (PSR) model was used to describe the plastic-elastic behavior of the tested materials, as well as to detect the presence of ISE. It was found that the increase in chromium concentration in the tested area was accompanied by a greater tendency to elastic deformation during nanoindentation.

## 1. Introduction

Boriding of nickel-based alloys causes the formation of a multiphase layer which differs in phase composition depending on the chemical composition of the substrate material. The most popular group of nickel alloys are superalloys containing chromium, cobalt, molybdenum or iron as the main alloying elements. As a result of boriding of nickel-chromium alloys, a layer composed of various types of nickel borides and chromium borides is formed. The gas-borided layers formed on the Inconel 600-alloy and Nimonic 80A-alloy contained a mixture of NiB, Ni_2_B, Ni_3_B, Ni_4_B_3_, CrB and Cr_2_B [1,2,3]. This characteristic fine-grained microstructure was characterized by the absence of phase zonation. The different types of nickel borides and chromium borides were observed only as a mixture. All phases appeared alternately across the entire boride layer, therefore a significant fluctuation of the concentration of nickel and chromium was observed on the cross-section of the borided layer [1,2,3]. In the case of the boride layer containing both nickel and chromium borides, the hardness ranged from 19.95 to 36.26 GPa. A similar effect was obtained for the Young’s modulus [2]. The highest values of hardness (36.26 GPa) and Young’s modulus (354.76 GPa) corresponded to the microstructure consisting only of a mixture of chromium borides (CrB + Cr_2_B). Simultaneously, the increase in the percentage of nickel borides in the tested area resulted in a decrease in hardness and elastic modulus [2]. The appropriate technique used to determine the hardness and Young’s modulus of a fine-grained microstructure consisting of various types of borides is nanoindentation. This method of measurements allows for a detailed analysis of the mechanical properties in the nanoscale for various types of borides. Nanoindentation is often used to determine the properties of iron borides [4,5,6], nickel and chromium borides [2,3], carboborides [7] or nitrides [8]. For a single-phase materials or layers, some authors report an indentation size effect (ISE). Generally, the indentation size effect can be defined as an increase in hardness (or Young’s modulus, strength) with decreasing indentation depth [9]. The occurrence of ISE may be related to several factors: friction, lack of measurement capabilities, the presence of surface oxides or chemical contamination, dislocation mechanisms [10]. The possibility of occurrence of an indentation size effect in the case of multiphase materials or layers has rarely been reported in the literature. The tendency to the indentation size effect in cermets was described in paper [11]. Titanium carbide cermets with different binder content were investigated. ISE was analyzed using Meyer’s law and the proportional resistance specimen model. It was found that the hardness decreases with increasing applied load, especially for lower loads. Moreover, fine-grained cermets showed a more pronounced tendency towards ISE [11]. Such conclusions inspired the analysis of the tendency to the indentation size effect in multiphase borided layers.

## 2. Materials and Research Methods

### 2.1. Production of a Multiphase Boride Layer

The multiphase boride layer was produced on a nickel-chromium alloy (Inconel 600). This material contained 15.72% Cr, 0.16% Mn, 0.04% Cu, 8.63% Fe, 0.18% Si, 0.078% C, 0.06% Al, and 75.132% Ni. The boriding method selected to produce the thick layer was gas boriding in N_2_-H_2_-BCl_3_ atmosphere. The continuous process was carried out at the temperature of 1193 K. The gas source of boron used in this process was boron trichloride with a concentration of 1.36 vol.% in relation to the entire atmosphere used (N_2_-H_2_-BCl_3_). After 2 h of boriding, the specimens were cooled under a protective nitrogen atmosphere.

### 2.2. Microstructure Characterization

The first investigation carried out immediately after gas boriding was the phase analysis. An EMPYREAN X-ray diffractometer (Malvern Panalytical Ltd., Malvern, UK) was used for this study. X-ray diffraction patterns (XRD) were measured using Cu K_α_ radiation. The samples for microstructure analysis and nanoindentation required metallographic preparation including mounting in a conductive resin, grinding, polishing and etching with Marble’s reagent. The microscopic observations and EDS microanalysis were performed using a Vega 5135 Scanning Electron Microscope (TESCAN, Brno, Czech Republic). After the nanoindentation measurements finished, the tested areas were examined in a respect of their chemical composition. The content of the main elements (nickel, chromium, iron and boron) occurring in the borided layer, were measured using an Avalon X-ray microanalyzer (Princeton Gamma Tech, Princeton, NJ, USA) equipped with an energy dispersive spectrometer (EDS).

### 2.3. Nanoindentation Experiments

Nanoindentation tests were performed using the NHT^3^ nanoindenter (Anton Paar, Warsaw, Poland) equipped with a Berkovich diamond tip. The measurements were carried out on the cross-section of the borided layer in areas with different amounts of chromium borides. Due to the characteristic fine-grained microstructure of the gas-borided layer formed on the Inconel 600-alloy, it was necessary to analyze the chemical composition of the tested areas. It was impossible to determine the amount of chromium borides (CrB and Cr_2_B mixture) and nickel borides (NiB, Ni_2_B, Ni_3_B and Ni_4_B_3_ mixture). However, it is obvious that the increase in chromium concentration in the examined area, accompanied by an increased boron content, indicated the presence of chromium borides. For this reason, the increase in chromium content (measured by EDS method) was related to the increase in the amount of chromium borides mixture in the examined areas. Based on the results of EDS microanalysis, the testing areas were classified into three groups according to their chromium content: 10 wt.%, 20 wt.% and 30 wt.%. It was assumed that the increase in the concentration of chromium was accompanied by an increase in the amount of chromium borides in the microstructure. For each group, measurements were carried out using indentation loads ranging from 10 mN to 500 mN.

Five measurements were performed for each indentation load. During the measurements, the load-displacement curves were automatically recorded and analyzed according to the Oliver-Pharr procedure [12]. On the basis of the load-displacement curve, the following parameters were determined: maximum load P_max_, maximum indentation depth h_max_, permanent indentation depth h_p_, tangent indentation depth h_r_, contact depth of the indenter with the sample at maximum load h_c_, contact stiffness S. The dependence between the indentation load and the indentation depth can be expressed by the following Equation:

(1) $$P=P_{\max}{\left(\frac{h-h_{p}}{h_{\max}-h_{p}}\right)}^{m}$$

where: P_max_—maximum load, h_max_—maximum indentation depth, h_p_—permanent indentation depth, m—empirically determined fitting parameter.

The nanoindentation hardness H_IT_ is defined as the ratio of the maximum indentation load P_max_, to the projected contact area A_p_ of the hardness impression, as follows:

(2) $$H_{\mathrm{IT}}=\frac{F_{\max}}{A_{p}}$$

where: P_max_—maximum load, A_p_—projected contact area.

The nanoindentation Young’s modulus E_IT_ depended on the plane-strain modulus E* and the Poisson’s ratio ν_s_, according to the Equation:

(3) $$E_{\mathrm{IT}}=E^{*}\left(1-\upsilon_{s}^{2}\right)$$

where: E*—plane-strain modulus, ν_s_—Poisson’s ratio of tested material.

After the nanoindentation measurements, the tested areas were examined in a respect of their chemical composition. The content of the main elements (nickel, chromium, iron and boron) present in the tested areas was measured using the energy dispersive spectrometer (EDS)-equipped PGT Avalon X-ray microanalyzer.

## 3. Experimental Results and Discussion

### 3.1. Microstructure of Borided Layer

**Figure 1:**
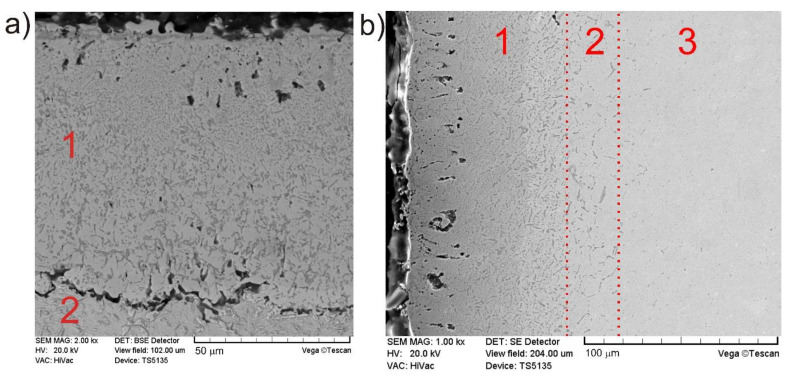
SEM microstructure of the gas-borided Inconel 600-alloy: (**a**) sample etched with Marble’s reagent, (**b**) sample without etching; (1)—compact zone of borides, (2)—zone with borides at the grain boundaries, (3)—substrate material.

SEM micrographs of the cross-sections of the borided Inconel 600-alloy are presented in Figure 1a,b. The produced compact boride layer (1) was characterized by a fine-grained microstructure with clearly visible two types of grains differing in morphology and color. Beneath the compact boride layer, the area with the borides at the grain boundaries was marked as (2). The presence of chromium and nickel borides at the grain boundaries was described and proved in the previous work [1]. The occurrence of zone with borides at grain boundaries beneath the compact boride zone indicates the type of the boron diffusion mechanism into the substrate material. Probably, the predominant mechanism consisted in the diffusion along the grain boundaries [1]. Zones marked as (1) and (2) can be collectively referred to as the diffusion layer. Such a layer is a result of diffusion of boron atoms into the workpiece, creating new phases that differ in structure and properties compared to the substrate material (3). The good quality of the produced layer can be considered in terms of the presence of small amount of porosity, which was observed near the top surface. It was a result of a formation of iron chlorides during the reaction between boron trichloride (from the gaseous atmosphere) and iron (from the substrate material). The second factor indicating the good quality of the gas-borided layer produced on Inconel 600-alloy can be the morphology of the boundary between the compact boride layer and the sublayer (zone with borides at the grain boundaries), as well as the smooth transition between the zone with borides at the grain boundary and the substrate material. In order to avoid the influence of etching on the morphology of these boundaries, microstructural observations were carried out on the non-etched sample (Figure 1b). The presence of porosity was not detected in the area at the boundaries between the analyzed zones. There were no visible signs of delamination of the compact boride layer from the substrate. In the case of observation carried out using the etched sample, it could appear that the layer is peeling off the substrate material (Figure 1a). However, such a situation was the result of etching with Marble’s reagent, which is a corrosive medium.

The presence of alloying elements (especially chromium) in the Inconel 600-alloy caused that the compact boride layer shows no needle-like morphology. The smooth morphology of the boundary between the compact boride layer and the material underneath them resulted from the presence of chromium atoms, which constituted a diffusion barrier that hindered the diffusion of boron atoms into the substrate material [13,14,15,16,17]. The average thickness of the compact zone of borides was 86 µm. Whereas, the average depth of the entire diffusion layer was 112 μm.

**Figure 2:**
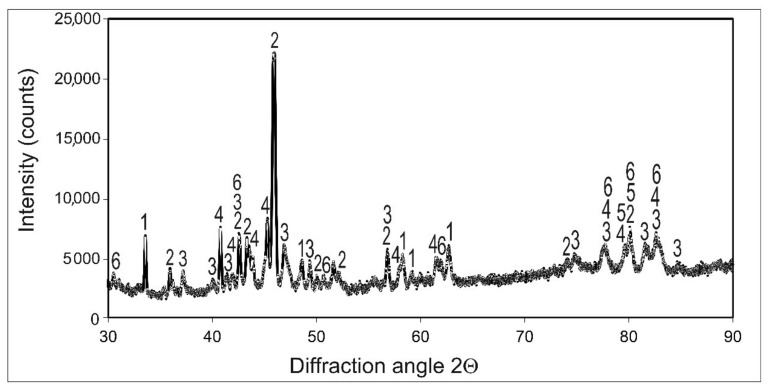
XRD patterns of gas-borided Inconel 600-alloy; 1—NiB, 2—Ni_2_B, 3—Ni_3_B, 4—Ni_4_B_3_, 5—CrB, 6—Cr_2_B.

Borided layer formed on Inconel 600-alloy using gas boriding in N_2_-H_2_-BCl_3_ atmosphere consisted of two types of grains (Figure 1a). However, in order to identify the indicidual phases and their distribution on the cross-section of the borided layer, XRD analysis and EDS microanalysis were necessary. Due to the high concentration of chromium (15.72 wt.%) in Inconel 600-alloy, the presence of chromium borides in the borided layer was expected. The XRD patterns shown in Figure 2 confirmed the presence of boron-rich nickel borides (NiB, Ni_4_B_3_) and boron-poor nickel borides (Ni_2_B, Ni_3_B), as well as chromium borides (CrB, Cr_2_B). The identified phases differed in peak intensities. The peaks corresponding to nickel borides dominated in the XRD patterns.

**Figure 3:**
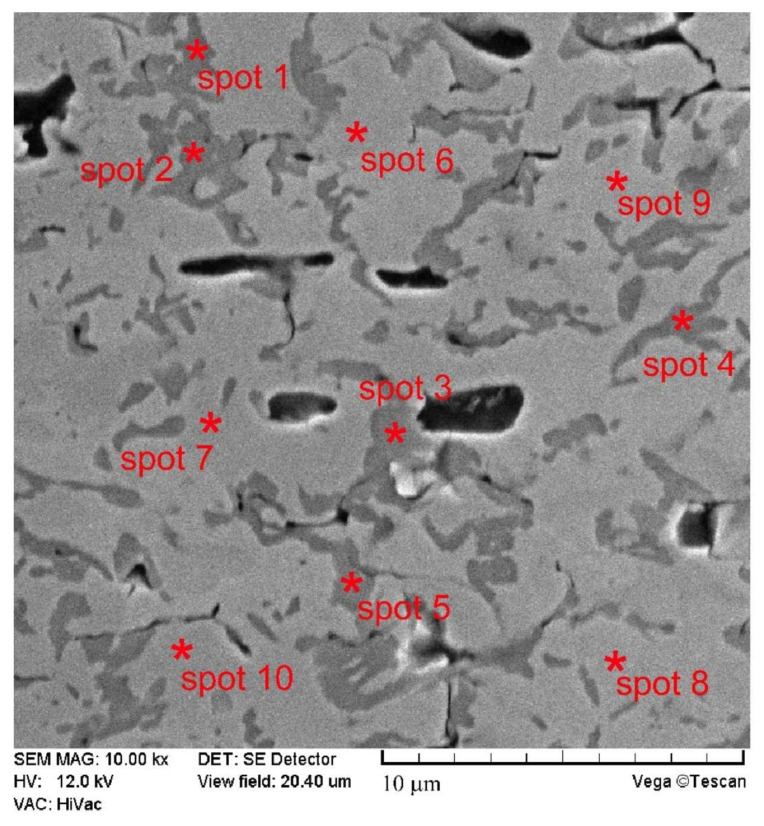
SEM microstructure of the gas-borided Inconel 600-alloy with marked spots in which EDS point microanalysis was performed and results of EDS point microanalysis; *—marking the exact point of the measurement; spots 1–5—the measurements carried out in the chromium borides mixture; spots 6–10—the measurements carried out in the nickel borides mixture.

Observation of the microstructure of the compact boride layer at higher magnifications (Figure 3) allows one to detect the presence of two different types of grains that differ visually in color. The results of the phase analysis confirmed the presence of nickel borides (NiB, Ni_4_B_3_, Ni_2_B, Ni_3_B) and chromium borides (CrB, Cr_2_B) in the produced layer. Hovewer, the distribution of each type of borides on the cross-secion of borided layer required the use of EDS microanalysis. The chemical composition of ten spots located in different areas of the layer was examined. The microstructure of the borided layer with marked test spots and areas were presented in Figure 3. The results of EDS microanalysis were presented in Table 1. According to the obtained results of point microanalysis presented in Table 1, it was found that the dark grains (spots 1–5) contained a high content of chromium and increased concentration of boron. Simultaneously, these grains contained a low concentration of nickel and iron. This indicated the presence of a mixture of chromium borides (CrB, Cr_2_B) in these grains. In the case of the results obtained from the light area (spots 6–10), an increased nickel concentration was observed, as well as a relatively low chromium content. Such a chemical composition indicated the presence of nickel borides (NiB, Ni_4_B_3_, Ni_2_B, Ni_3_B) in these grains. Generally, it was impossible to determine the distribution of indicidual phase in the microstructure. Even at high magnification, it was impossible to identify a separate type of chromium borides (CrB, Cr_2_B) in the darker area. Taking into account the chemical formula of individual chromium borides, the EDS results obtained do not match the specific phase. The theoretical contents of chromium and boron in chromium borides differ from the results presented in Table 1. This indicates that the chromium borides only occurred as a mixture. Similarly, in the case of light areas, the identification of a separate type of nickel borides (NiB, Ni_4_B_3_, Ni_2_B, Ni_3_B) was also difficult. Based on the EDS microanalysis results, it was found that nickel borides (all four types) and chromium borides (two types) exist only in the form of a fine-grained mixture.

**Figure 4:**
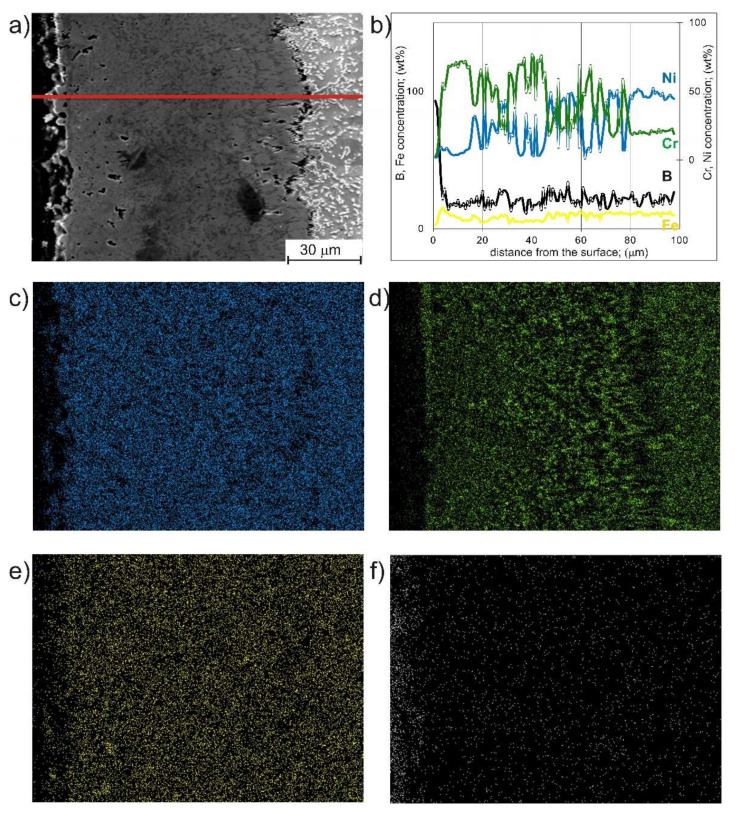
SEM microstructure of the gas-borided Inconel 600-alloy with a marked line along which EDS microanalysis was performed (**a**), the results of the linear EDS microanalysis (**b**) and the EDS maps obtained for nickel (**c**), chromium (**d**) iron (**e**) and boron (**f**).

It was visible that the distribution of nickel borides and chromium borides on the cross-section of the compact boride layer was irregular, without clear zonation. In order to confirm the occurrence of this characteristic phase distribution in the microstructure, a linear EDS analysis and EDS maps were performed (Figure 4). The results of the linear EDS microanalysis (Figure 4b) showed high fluctuations in the concentration of nickel and chromium on the cross-section of the borided layer. A characteristic tendency was observed for a simultaneous high chromium content with a low nickel content. These results confirmed that nickel borides and chromium borides appear alternately on the cross-section of the borded layer. The same tendency is noticeable on EDS maps (Figure 4c–f).

### 3.2. Nanomechanical Properties of Borided Layer

**Figure 5:**
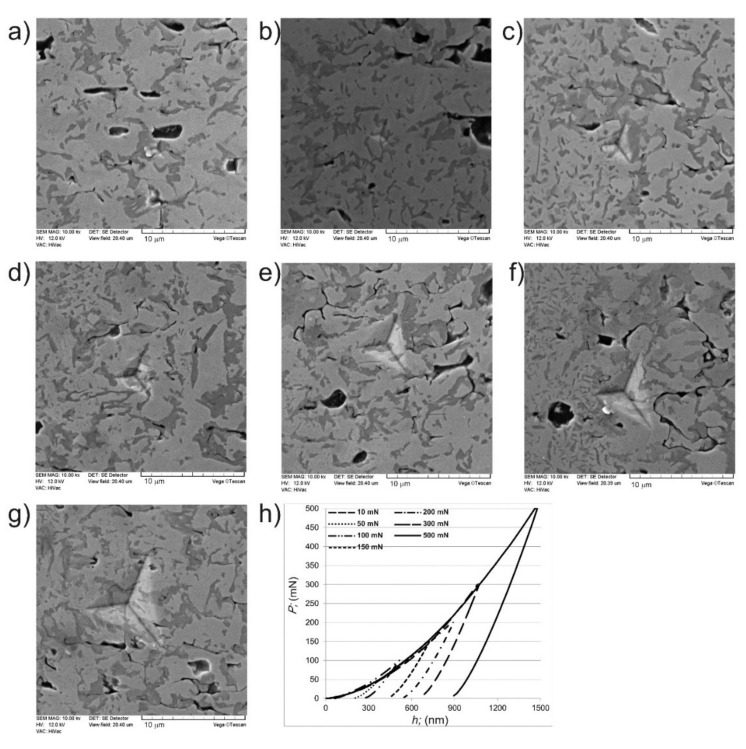
SEM images of areas with 20 wt.% of chromium with visible indents performed after nanoindentation under a maximum load of (**a**) 10 mN, (**b**) 50 mN, (**c**) 100 mN, (**d**) 150 mN, (**e**) 200 mN, (**f**) 300 mN, (**g**) 500 mN and (**h**) load–displacement curves recorded for areas with 20 wt.% of chromium during nanoindentation under a load range from 10 mN to 500 mN.

Berkovich nanoindentation was used to determine the parameters necessary to analyze the presence of the indentation size effect in the borided layer. Figure 5a–g shows representative indents obtained after nanoindentation under a load of 10–500 mN performed in the tested areas with 20 wt.% of chromium. Simultaneously, the load-displacement curves recorded during these measurements are shown in Figure 5h. It was obvious, that the increasing indentation load caused an increase in the dimensions of the indents (Figure 5a–g). All indents were produced in the areas with a combined microstructure containing nickel borides and chromium borides. The average concentration of chromium in the studied areas presented in Figure 5a–g was 20 wt.%, therefore the visual effect of such a chemical composition was the predominant amount of light grains in the SEM micrographs. These grains were identified by point analysis as a mixture of nickel borides.

Important information can be obtained from the shape of the load-displacement curves (Figure 5h). Generally, if the material represents fully elastic deformation, the unloading part of the load-displacement curve would lie on the loading part. Whereas, the tendency to the full plastic deformation during nanoindentation will be visible on the load-displacement curve as an unloading part of the curve perpendicular to the displacement axis. The shape of the load−displacement curves recorded for all indentation loads applied during the measurements indicated a tendency for mixed plastic and elastic deformation of the tested material during nanoindentation (Figure 5h). This is indicated by the fact that the loading curve differed significantly from the unloading curve. In this case, the area enclosed between the loading and unloading part of the curve represented the energy lost during plastic deformation. The increase in the indentation load was accompanied by an increase in the maximum penetration depth h_max_ (Figure 5h).

**Figure 6:**
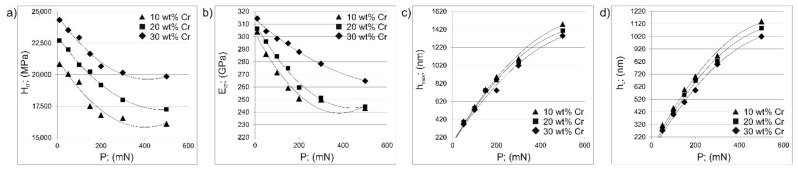
Dependence of the indentation hardness H_IT_ (**a**), indentation elastic modulus E_IT_ (**b**), maximum penetration depth h_max_ (**c**) and contact depth of the indenter with the sample at maximum load h_c_ (**d**) on the indentation load P obtained for testing areas differing in the concentration of chromium.

Three types of areas differing in chromium concentration were examined. The average values of H_IT_, E_IT_, h_max_ and h_c_ were calculated and are presented in Table 2, as well as in Figure 6. When comparing the average values of penetration depths obtained for areas differing in chromium concentration, significant differences were visible. In the case of such a layer with a multiphase microstructure, the amount of chromium borides and nickel borides strongly influenced the properties of the tested areas. It was earlier reported [2,3] that the percentage of chromium borides increases the nanomechanical properties. Therefore, in the present study, the increase in chromium concentration in the studied areas resulted in lower values of the penetration depths h_max_ and h_c_. As a consequence, areas with higher Cr content were characterized by higher indentation hardness H_IT_ and indentation elastic modulus E_IT_. A comparison of the indentation hardness and the elastic modulus obtained under different indentation loads shows significant differences. An increase in the indentation load from 10 mN to 500 mN resulted in a decrease in hardness H_IT_ and Young’s modulus E_IT_. In the case of the tested areas with the lowest chromium concentration (10 wt.%), the average decrease in H_IT_ and E_IT_ was 23% and 20%, respectively, comparing the average values obtained for the indentation loads of 10 mN and 500 mN. The increase in the chromium concentration in the tested areas was the cause of the percentage decrease in the indentation hardness and the decrease in the elastic modulus obtained at the indentation loads of 10 mN and 500 mN, 21% and 18% for tested areas with 20 wt.% of Cr, 19% and 16% for tested areas with 30 wt.% of Cr. This situation suggested the presence of the indentation size effect.

### 3.3. Indentation Size Effect (ISE) Analysis

In general, the indentation size effect (ISE) phenomenon determines the tendency to the increase in indentation hardness with decreasing indentation load. This situation is related to the nucleation of the dislocations in the plastic zone under the indenter pressure. The presence of dislocations in the plastic zone increases the effective yield strength of the tested material, and thus the increase in hardness is observed [9]. Susceptibility to the indentation size effect (ISE) can be determined by the following models: Meyer’s law, the proportional specimen resistance (PSR) model, or the elastic recovery (ER) model.

#### 3.3.1. Meyer’s law

The ISE phenomenon can be described by the Meyer’s law, as follows [4,18]:

(4) $$P_{\max}=A\cdot h_{c}^{n}$$

where: P_max_—maximum indentation load, h_c_—contact depth of the indenter with the sample at maximum indentation load, A—constant derived from the curve fit of the plot on a bilogarithmic scale, n—Meyer’s index.

This equation shows the relationship between the applied indentation load P_max_ and the size of indent, represented by the contact depth of the indenter with the sample at maximum indentation load h_c_. The value of the Meyer’s index n indicates whether the tested material has a tendency to ISE. In the case where the value of the exponent n is less than 2, an indentation size effect occurs, especially in hard, brittle materials such as ceramic phases. The exponent n equal to 2 indicates the independents of the indentation hardness from the indentation load. When the Meyer’s index is greater than 2, the reverse of the ISE behavior appears [4,18].

**Figure 7:**
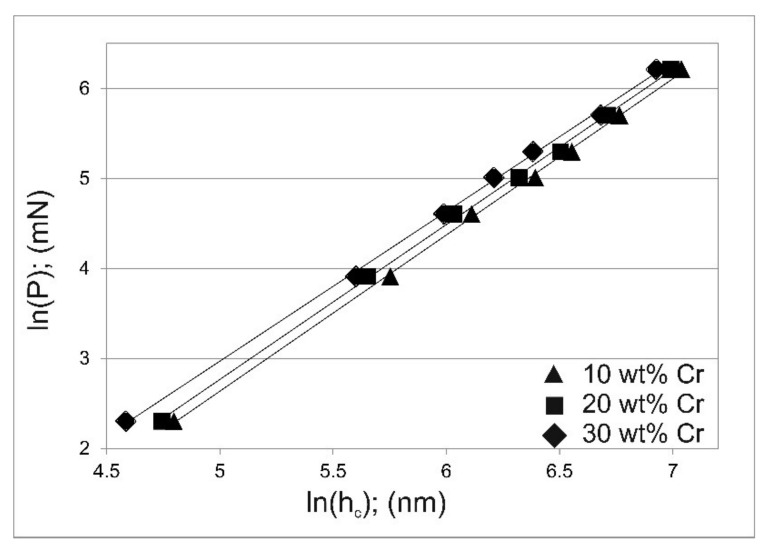
Dependence of the indentation load P on the contact depth of the indenter with the sample at maximum indentation load h_c_ on a bilogarithmic scale according to Meyer’s law for the tested areas containing 10, 20 and 30 wt.% of Cr.

The applied indentation loads from 10 mN to 500 mN and the average values of the contact depth of the indenter with the sample at maximum indentation load (h_c_) were presented in a bilogarithmic scale according to Meyer’s law. The ln(P)/ln(h_c_) dependence obtained for the tested areas containing 10 wt.% of Cr, 20 wt.% of Cr, and 30 wt.% of Cr is shown in Figure 7. In all cases, the plot showed a linear dependence, which indicates that the traditional Meyer’s law is appropriate to describe the obtained nanoindentation results. Based on the linear regression analyzes of the obtained plots on the bilogarithmic scale (Figure 7), the best-fit Meyer’s index values were determined. In all cases, the Meyer’s index was less than 2: 1.737 for the tested areas containing 10 wt.% of chromium, 1.722 for the tested areas containing 20 wt.% of chromium, 1.664 for the tested areas containing 30 wt.% of chromium. These results confirmed the occurrence of the indentation size effect phenomenon in the gas-borided layers produced on the Inconel 600-alloy. Simultaneously, it can be seen that the increase in chromium concentration in the tested area was accompanied by a decreasing Meyer’s index. This indicates that a higher concentration of chromium, and thus an increased percentage of chromium borides in the microstructure, resulted in a greater tendency to the ISE phenomenon occurrence.

#### 3.3.2. PSR model

An alternative proposal for ISE analysis is the proportional specimen resistance (PSR) model. In this model, it is assumed that the resistance of the tested materials to the permanent deformation is not constant, but increases linearly with the size of the indent [18,19].

The basis of this model was taken from the concept developed by Hays and Kendall [20], which assumed that there exists a minimum value of the indentation load (effective indentation load), below which there is no permanent deformation, but only an elastic deformation occurs [18,21]. The value of the effective indentation load P_eff_ can be calculated by plotting the experimental data for the indentation load P as a function of the square of the contact depth h_c_. As a consequence, the effective indentation load P_eff_ can be expressed by the following equation:

(5) $$P_{\mathrm{eff}}=P-W=K\cdot h_{c}^{2}$$

where: W—material resistance to the initiation of plastic flow, K—constant, h_c_—contact depth of the indenter with the sample at maximum indentation load.

**Figure 8:**
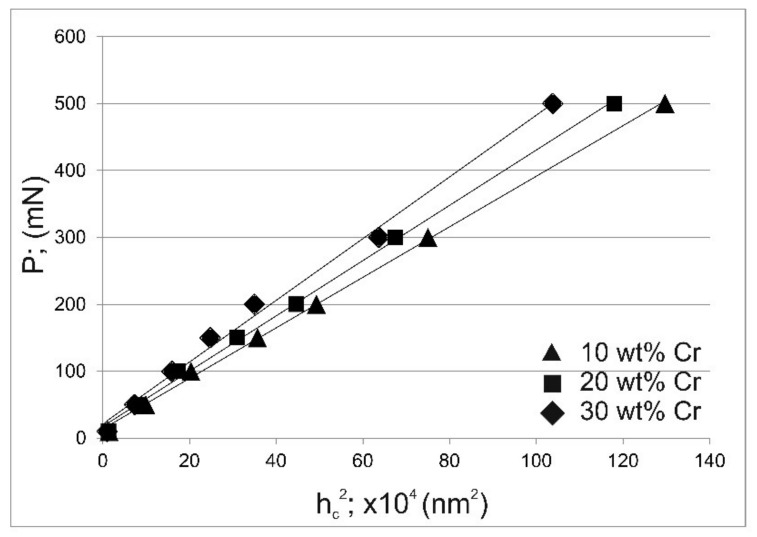
Dependence of the indentation load P on the square of the contact depth of the indenter with the sample at maximum indentation load h_c_ according to Hays and Kendall model for the tested areas containing 10, 20 and 30 wt.% of Cr.

P vs. h_c_^2^ plots are presented in Figure 8 for the tested areas containing 10 wt.%, 20 wt.% and 30 wt.% of chromium. The most important parameter that can be calculated from Figure 8 was the parameter W. This constant is the minimum indentation load at which the material would exhibit permanent (plastic) deformation. The estimated best-fit values of W were as follows: 13.7, 17.5 and 21.5 mN, for 10 wt.%, 20 wt.% and 30 wt.% of Cr, respectively. The lowest indentation load used in this study was 10 mN, which was a value lower than that calculated as a W parameter for all tested areas. The shape of the load-displacement curves indicated that the permanent deformation had already occurred with an applied load of 10 mN. Simultaneously, it can be concluded that the minimum indentation load necessary to initiate permanent deformation is lower than 10 mN. This means that the determined parameter W does not provide correct information about the ISE phenomenon.

In the PSR model proposed by Li and Bradt [19] it was suggested that the value of W is proportional to the indentation size and should be written as follows:

(6) $$W=a_{1}\cdot h_{c}$$

Simultaneously, the effective indentation load can be expressed as:

(7) $$P_{\mathrm{eff}}=P-W=a_{2}\cdot h_{c}^{2}$$

or:

(8) $$P=a_{1}\cdot h_{c}+a_{2}\cdot h_{c}^{2}$$

where: a_1_, a_2_— constants determined by the polynomial regression.

When Equation (8) was modified by the linear regression in the form of $\frac{P}{h_{c}}$ versus h_c_, the following equation was obtained:

(9) $$\frac{P}{h_{c}}=a_{1}+a_{2}\cdot h_{c}$$

**Figure 9:**
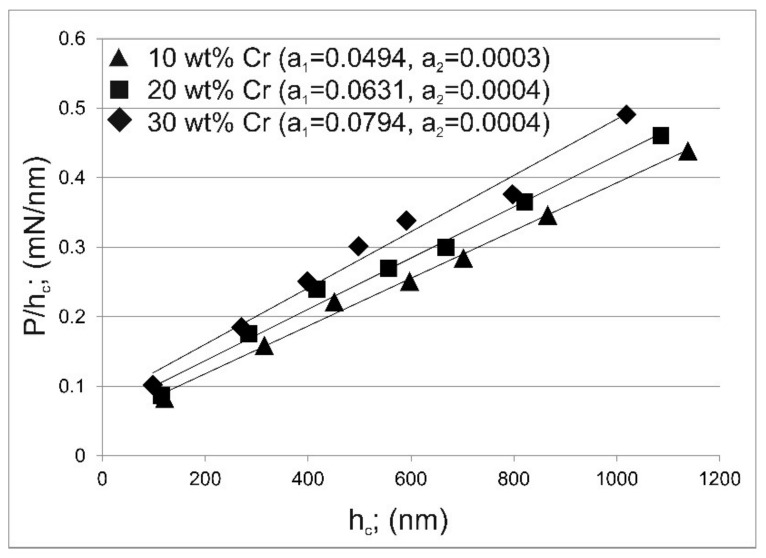
Dependence of the $\frac{P}{h_{c}}$ on the contact depth of the indenter with the sample at maximum indentation load h_c_ according to Li and Bradt model for the tested areas containing 10, 20 and 30 wt.% of Cr.

The dependence of the $\frac{P}{h_{c}}$ versus h_c_ is shown in Figure 9 for areas differing in chromium concentration. The calculated values of the constants a_1_ and a_2_ are also presented. The positive values of the parameter a_1_ indicate that the PSR model is appropriate for describing the indentation size effect for the tested material. In the case of a negative value of a_1_, it is clear that the PSR model cannot provide a satisfactory explanation of the ISE observed for the tested material [18]. For each studied area, the value of a_1_ parameter was higher than 0 (Figure 9), therefore it was found that the proportional specimen resistance (PSR) model is suitable for describing the ISE phenomenon for the gas-borided layer produced on Inconel 600-alloy.

In general, the parameters a_1_ and a_2_ calculated on the basis of Figure 9 can also be used to express the elastic and plastic portion of the energy consumed during nanoindentation [18,19,20,21]. This means that the parameter a_1_ can be used to describe the level of elastic properties (represented, for example, by the Young’s modulus). In the case of the gas-borided layer produced on Inconel 600-alloy, the following values of a_1_ parameter were obtained: 0.0494 for tested area containing 10 wt.% of chromium, 0.0631 for tested area containing 20 wt.% of chromium, 0.0794 for tested area containing 30 wt.% of chromium. This general tendency, showing that with the increasing chromium concentration in the tested area, the parameter a_1_ increased, was in good agreement with the information provided from the load-displacement curves. The position of the unloading curve on the load-displacement curve indicated that the increased concentration of chromium caused that the measured area was more prone to elastic deformation than plastic (permanent) deformation. In the case of the PSR model proposed by Li and Bradt, good agreement was achieved with the obtained nanoindentation results. Therefore, this model is suitable for describing the ISE phenomenon in the gas-borided layers produced on Inconel 600-alloy.

#### 3.3.3. Geometrically Necessary Dislocation Model

From a microstructural point of view, indentation size effect phenomenon is attributed to the evolution of geometrically necessary dislocations (GNDs) beneath the Berkovich indenter. The presence of dislocations caused an increase in the strain gradients, and thus the formation of a plastically deformed zone [22,23,24]. Nix and Gao [22] showed that the density of geometrically necessary dislocations created within the plastic zone bounded by the circle of contact for a conical indenter can be determined based on the indentation depth dependence of the hardness. This model was used to describe the ISE phenomenon in various materials [23,24,25]. The density of the geometrically necessary dislocations increases with the decrease of the indentation depth h. This allows the hardness to be expressed as H_0_, which would be obtained without the presence of the geometrically necessary dislocations, according to the following formula:

(10) $$\frac{H}{H_{0}}=\sqrt{1+\frac{h^{*}}{h}}$$

where: h*—characteristic length scale that is dependent on the indenter shape and material properties, H—indentation dependent hardness, H_0_—the indentation hardness at large indentation depths.

H_0_ parameter is the hardness in the limit of infinite depth, which can be estimated by extrapolating the linear relations of H^2^ against 1/h [22]. Whereas, parameter h* can be expressed as [24]:

(11) $$h*=\zeta \hat{l}$$

where: $\hat{l}$—intrinsic material length scale, ζ—length scale parameter given by [24]:

(12) $$\zeta =\frac{3}{2c\bar{r}}\;\tan\theta$$

where: θ—contact angle of the Berkovich indenter with the surface, c—material constant, $\bar{r}$—Nye’s factor, which is a scalar measure of GNDs [24].

**Figure 10:**
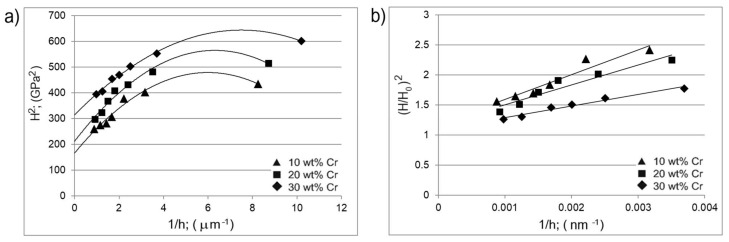
Dependence of the hardness on depth according to the Nix and Gao model: (**a**) the dependence H^2^ to 1/h, (**b**) the dependence (H/H_0_)^2^ to 1/h for the tested areas containing 10, 20 and 30 wt.% of Cr.

In order to determine the H_0_ and h* parameters, graphs of the dependence H^2^ to 1/h, as well as (H/H_0_)^2^ to 1/h were plotted (Figure 10). All the determined parameters necessary to describe the geometrically necessary dislocation model are presented in Table 3.

In general, the lower dimensions of the indent are accompanied by a greater strain gradient and a higher density of geometrically necessary dislocations (GNDs) [24]. In the case of gas-borided layer produced on Inconel 600-alloy, a strong effect of chromium concentration on H_0_, h* and $\hat{l}$ was observed. The increase in hardness in the tested areas with higher chromium content resulted in a decrease in h* and $\hat{l}$. In the case of tested area containing 30 wt.% of chromium, the lowest length scale parameters were calculataed ($\hat{l}$ = 0.206). This situation indicated that the material of higher hardness is more susceptible to the indentation size phenomenon [24]. It was suggested by some authors [24,26] that the length scale parameter $\hat{l}$ is proportional to the mean distance between the statistically stored dislocation. The lower value of length scale parameter can be related to the reduced distances between the statistically stored dislocations. The presence of statistically stored dislocation and dislocations created by the impression of the indenter resulted in an increase in the effective yield strength of the material, and thus increase in hardness.

## 4. Summary and Conclusions

The nanomechanical properties of the multiphase gas-borided layer formed on Inconel 600-alloy were investigated using a Berkovich diamond tip. To determine the dependence of nanomechanical properties on the applied load, the indentation load range from 10 mN to 500 mN was used. The presence of the indentation size effect (ISE) was analyzed using four models: Meyer’s law, Hays and Kendall model, Li and Bradt model, Nix and Gao model. The tested areas were selected into three groups differing in the concentration of chromium. The increase in chromium concentration in the tested area was accompanied by an increase in indentation hardness and Young’s modulus. The analysis of the dependence of the load on nanomechanical properties showed that the increase in the indentation load resulted in a decrease in the indentation hardness and Young’s modulus.

For all examined areas, the calculated Meyer’s index was lower than 2, therefore it can be concluded that the Meyer’s law can be used as a method of ISE detection in the gas-borided layer. Simultaneously, it can be seen that the increase in chromium concentration in the tested area was accompanied by a decreasing Meyer’s index. This indicates that a higher concentration of chromium, and thus an increased percentage of chromium borides in the microstructure, resulted in a greater tendency to the ISE phenomenon.

Thd Hays and Kendall model was used to calculate the minimum indentation load (W) at which the material would exhibit permanent (plastic) deformation. The obtained experimental results were not consistent with the theory of Hays and Kendall. Simultaneously, the PSR model developed by Li and Bradt was used to describe the plastic-elastic behavior of the tested materials, as well as to detect the presence of ISE. It was found that the increase in chromium concentration in the tested area was accompanied by a greater tendency to elastic deformation during nanoindentation. Nix and Gao model applied to describe the role of dislocation in the occurrence of ISE confirmed that the tested areas with a higher chromium concentration were more susceptible to the ISE phenomenon.

## Figures and Tables

**Table 1:** The results of point EDS microanalysis (wt.% of Cr, Ni, B and Fe) taken from spots marked in Figure 3.

Element	Spot 1	Spot 2	Spot 3	Spot 4	Spot 5	Spot 6	Spot 7	Spot 8	Spot 9	Spot 10
Cr	80.11	85.08	82.47	77.32	83.85	4.60	4.98	6.45	3.14	5.78
Ni	3.45	2.88	5.07	4.14	4.84	79.49	83.14	76.32	85.77	78.42
B	14.40	8.45	9.74	15.88	9.16	8.55	7.32	10.14	7.07	10.21
Fe	2.04	3.59	2.72	2.66	2.15	7.36	4.65	7.09	4.02	5.59

**Table 2:** The results of the nanoindentation measurements carried out under a load of 10–500 mN obtained in the borided layer in the measuring area containing 10, 20 and 30 wt.% of chromium.

		Indentation Load						
		10 mN	50 mN	100 mN	150 mN	200 mN	300 mN	500 mN
H_IT_ (MPa)	10 wt.% Cr 20 wt.% Cr 30 wt.% Cr	20847.2 22714.8 24331.7	20059.5 21981.6 23519.7	19437.1 20782.0 22929.0	17511.5 20223.3 21653.3	16798.4 19165.5 20650.4	16558.9 17989.9 20150.8	16109.8 17236.3 19849.3
E_IT_ (GPa)	10 wt.% Cr 20 wt.% Cr 30 wt.% Cr	303.9 306.2 314.4	286.2 296.2 304.2	271.4 284.4 298.4	259.2 274.8 294.6	250.8 259.4 287.8	250 251.2 278.4	243.2 244.4 264.8
h_max_ (nm)	10 wt.% Cr 20 wt.% Cr 30 wt.% Cr	158 153.8 149.8	398.8 386.4 365.8	559.6 541.4 525.4	752.4 739.47 39.4	891.6 857 741.2	1094 1058.8 1018	1479 1406.8 1349.2
h_c_ (nm)	10 wt.% Cr 20 wt.% Cr 30 wt.% Cr	120 114.2 97.4	315.2 285.6 270.8	451 416.6 399	597.6 556 498	702.2 667.4 591	866.2 821 798.2	1138 1086 1019.6

**Table 3:** Length scale parameters estimated based on Nix and Gao model from the fitted nanoindentation results obtained in the measuring area containing 10, 20 and 30 wt.% of chromium.

Chromium Content	H_0_ (GPa)	h* (nm)	$\hat{l}$ (µm)
10 wt.% Cr	12.894	411.99	0.442
20 wt.% Cr	14.625	329.91	0.354
30 wt.% Cr	17.638	192.49	0.206

## Data Availability

The authors confirm that all the data supporting the findings of this study is presented within the article.
